# DNA barcodes for Aotearoa New Zealand Pyraloidea (Lepidoptera)

**DOI:** 10.3897/BDJ.8.e58841

**Published:** 2020-11-27

**Authors:** Renate Wöger, Roland Wöger, Matthias Nuss

**Affiliations:** 1 Senckenberg Museum of Zoology, Dresden, Germany Senckenberg Museum of Zoology Dresden Germany

**Keywords:** Pyralidae, New Zealand, Crambidae, Scopariinae, COI, barcode, BIN, ABGD

## Abstract

Identification of pyraloid species is often hampered by highly similar external morphology requiring microscopic dissection of genitalia. This becomes especially obvious when mass samples from ecological studies or insect monitoring have to be analysed. DNA barcode sequences could accelerate identification, but are not available for most pyraloid species from New Zealand. Hence, we are presenting a first DNA-barcode library for this group, providing 440 COI barcodes (cytochrome C oxidase I sequences) for 73 morphologically-identified species, which is 29% of Pyraloidea known from New Zealand. Results are analysed using the Barcode Index Number system (BIN) of BOLD and the Automatic Barcode Gap Discovery method (ABGD).

Using BIN, the 440 barcodes reveal 82 clusters. A perfect match between BIN assignment and morphological identification was found for 63 species (86.3%). Four species (5.5%) share BINs, each with two species in one BIN, of which *Glaucocharis epiphaea* and *Glaucocharis harmonica* even share the same barcode. In contrast, six species (8.2%) split into two or more BINs, with the highest number of five BINs for *Orocrambus ramosellus*. The interspecific variation of all collected specimens of New Zealand Pyraloidea averages 12.54%. There are deep intraspecific divergences (> 2%) in seven species, for instance *Orocrambus vulgaris* with up to 6.6% and *Scoparia ustimacula* with 5.5%.

Using ABGD, the 440 barcodes reveal 71 or 88 operational taxonomic units (OTUs), depending on the preferred partition. A perfect match between OTU and morphological identification was found for 56 species (76.7%) or 62 species (84.9%). ABGD delivers four or seven species sharing OTUs and four or ten species split into more than one OTU.

Morphological re-examination, as well as the analysis of a concatenated dataset of COI and the nuclear markers EF1α and GADPH for species split into more than one BIN or OTU, do not support a higher number of species. Likewise, there is no evidence for *Wolbachia* infection as a trigger for these sequence variations.

## Introduction

The DNA barcode is a 658 bp mitochondrial cytochrome oxidase I gene (COI) sequence (Hebert 2003). It is generally suitable for species delimitation due to its relatively-low intraspecific and high interspecific sequence variation (Hebert et al. 2004). It has been used for different animal groups (e.g. Manfredini 2008, Ward 2009, Miller et al. 2013, Hendrich et al. 2014, Schmidt et al. 2017) and is an accepted tool for molecular species identification in Lepidoptera (e.g. Hausmann et al. 2013, Wilson et al. 2013, Huemer et al. 2020). There are several studies demonstrating the effectivity and efficiency of “barcoding” (e.g. Hebert et al. 2004; Armstrong and Ball 2005, Hajibabaei et al. 2007, Huemer et al. 2020). Limitations of this method for species identification have been discussed by, for example, Mitchell (2008), Krishnamurthy and Francis (2012) and Taylor and Harris (2012). Different analytical methods for DNA Barcodes data are compared by Kekkonen and Hebert (2014) and Huang et al. (2020).

Even though there has already been a great number of DNA barcode campaigns for Lepidoptera with an increasing number of barcode libraries (e.g. Hausmann et al. 2011, Wilson et al. 2013, Nieukerken et al. 2016, Huemer et al. 2020), there is still a lack of such a library for the Pyraloidea of New Zealand. There are 250 pyraloid species known from New Zealand and most of them are endemic to the country. A total of 232 species belong to Crambidae and 18 to Pyralidae (Nuss et al. 2020). Crambinae and Scopariinae are the two most speciose lineages with 81 and 129 species, respectively (Dugdale 1988, Nuss et al. 2020). Their larvae are mostly terrestrial, but Acentropinae are adapted to aquatic habitats. As far as is known, all New Zealand species are phytophagous in the larval stage, those of Crambinae and Scopariinae feeding on Poales and Bryophyta, respectively (Leger et al. 2019).

Taxonomically, the pyraloid fauna of New Zealand is well studied (Meyrick 1882, Meyrick 1883, Meyrick 1884, Meyrick 1885a, Meyrick 1885b, Meyrick 1885c, Meyrick 1888, Meyrick 1889, Meyrick 1897, Meyrick 1901, Meyrick 1905, Meyrick 1909, Meyrick 1911, Meyrick 1912, Meyrick 1913, Meyrick 1914, Meyrick 1915, Meyrick 1919, Meyrick 1920, Meyrick 1921, Meyrick 1923, Meyrick 1924, Meyrick 1926, Meyrick 1927, Meyrick 1929, Meyrick 1931, Meyrick 1937, Meyrick 1938, Philpott 1918, Philpott 1920, Philpott 1923, Philpott 1924, Philpott 1926, Philpott 1927, Philpott 1928, Philpott 1929a, Philpott 1929b, Philpott 1931, Hudson 1928, Hudson 1939, Gaskin 1971, Gaskin 1973, Gaskin 1974, Gaskin 1975) and an overview is available via a digital image gallery (Hoare 2020). Despite all these sources, the identification of moths remains time-consuming, based on external morphological characters if there are similar interspecific or distinct intraspecific wing patterns. Such a situation is repeatedly found, for example, in the genera *Orocrambus* and *Eudonia*, which requires genitalia dissection and thus hampers efficient identification of species. Since DNA barcoding could accelerate species identification, we are presenting a first step towards a DNA library for New Zealand Pyraloidea.

## Materials and methods

### Fieldwork

We surveyed Pyraloidea in New Zealand during January and February of the years 2017 and 2018. Moths were attracted to artificial UV light for 3–4 hours after nightfall. Each collecting locality has been visited one to six times, depending on travel logistics and weather conditions. The moths were collected at 12 sites, of which three sites are in the Taranaki region on the North Island and nine sites are scattered over the South Island. Specimens studied originate from different ecoregions like Podocarp forests and domains of horticulture on the North Island (Taranaki), as well as beech forest (Karamea), tussock grassland (Central Otago) and coastal shrub (Waikawa) on the South Island. The data record is biased towards man-made habitats, as well as geographically towards the South Island.

At each locality, all attracted pyraloids were collected. Specimens were killed using ammonia or ethyl acetate, pinned and dried for transportation.

### Species identification

Specimens were identified by the authors using the database of Landcare Research Auckland (Hoare 2020) and the revision of the genus *Orocrambus* by Gaskin (1975). These resources are based on the morphology of external and genitalia characters.

Nomenclature and taxonomy are based on the Global Information System on Pyraloidea (GlobIZ) (Nuss et al. 2020). In cases where wing pattern elements are not sufficient for species identification, genitalia dissections were made following the protocols by Robinson (1976) and Nuss (2005).

### DNA extraction, PCR and sequencing

After fieldwork, collected moths were labelled and sorted to morpho-species. Species with deep morphological variation were additionally sorted into morpho-groups. One to three specimens, depending on the number of available specimens, of every group of unambiguously-identified species and every morpho-group, were chosen for DNA barcoding. DNA barcodes were obtained from the collected material and additionally from loaned specimens from Landcare Research Auckland, New Zealand.

Genomic DNA was extracted from dried abdomens by using the *Genomic DNA from tissue* kit (Macherey-Nagel, Germany), following the manufacturer‘s standard protocol for animal tissue.

Specimens older than 20 years were examined following the above-mentioned protocol under UV radiation to avoid DNA contamination.

Extracted DNA was used for amplifying the 5P fragment of the mitochondrial DNA cytochrome C oxidase I gene "barcoding region" (COI Barcode) via PCR with the primer combination HybHCO/HybLCO (Folmer et al. 1994; Wahlberg and Zimmermann 2005). These primers contain a universal primer tail (T7), which is also used for sequencing (Wahlberg and Wheat 2008). The PCR was performed in 20 µl reactions, containing 10 pmol of each primer, 10mM dNTPs, 2 µl PCR 10x OptiBuffer, 100mM MgCl_2_ and 0.5 U taq DNA Polymerase (BIORON GmbH Ludwigshafen). After an initial phase at 95ºC for 5 min the temperature profile was 95ºC for 30 sec, 50ºC for 30 sec and 72ºC for 45 sec for a total of 38 cycles. The final elongation temperature was 72ºC for 10 minutes followed by a cooling phase at 8ºC. To determine amplicon presence and size, we examined PCR results via gel electrophoresis on a 1% agarose gel and GelRed as dye agent.

For species split into more than one BIN, we amplified and sequenced the nuclear markers EF1α and GADPH.

We amplified EF1α PCR with the primer combination HybOskar (5' -TAA TAC GAC TCA CTA TAG GG GGC CCA AGG AAA TGG GCA AGG G-3')/HybEFrcM4 (5'-ACA GCV ACK GTY TGY CTC ATR TC-3') and GADPH PCR with the primer combination HybFrigga/Burre (Wahlberg and Wheat 2008). These primers contain a universal primer tail (T7), which is also used for sequencing (Wahlberg and Wheat 2008). The PCR was performed each in 20 µl reactions, containing 10 pmol of each primer, 10mM dNTPs, 2 µl PCR 10x GoldBuffer, 100mM MgCl_2_ and 0.1 U Amplitaq DNA Polymerase (Thermo Fisher Scientific GmbH, Dreieich). After an initial phase at 95ºC for 10 min, the temperature profile was 95ºC for 30 sec, 50ºC for 30 sec and 72ºC for 45 sec for a total of 40 cycles. The final elongation temperature was 72ºC for 8 minutes following by a cooling phase at 8ºC. To determine amplicon presence and size, we examined PCR results via gel electrophoresis on a 1% agarose gel and GelRed as dye agent.

For sequencing work, we mandated Macrogen Europe, Amsterdam, Netherlands.

### Data analysis

Sequences of COI, EF1α and GADPH were aligned manually using BioEdit version 7.2.6.1 (Hall 1999) and MEGA X version 10.1 (Kumar et al. 2018). For analysing the data, we used MEGA X, version 10.1 (Kumar et al. 2018) and the workbench supplied by the BOLD system (Ratnasingham and Hebert 2007). For analysis of the COI sequences, we used all specimens with a barcode sequence length > 500 bp which is regarded as a sufficient length for BIN assignment (Ratnasingham and Hebert 2013). The neighbour-joining method (Saitou and Nei 1987) was used to visualise similarity. Associated taxa were clustered with the bootstrap test with 1000 replicates (Felsenstein 1985). Evolutionary distances were calculated using the Kimura 2-parameter method (Kimura 1980). Minimum pairwise distance is shown for the genetic distance between species and maximum pairwise distance for intraspecific variation.

We analysed our data using the Barcode Index Number system (BIN) (Ratnasingham and Hebert 2013) and Automatic Barcode Gap Discovery (ABGD) (Puillandre et al. 2011). Both systems are two-phased and group specimens into Operational Taxonomic Units (OTU). The applied clustering algorithms and the initial threshold for the first OTU boundaries are the main differences between the two analysis methods. BINs were analysed using BOLD (Ratnasingham and Hebert 2007) for all sequences with more than 500 bp. ABGD analysis (Puillandre et al. 2011) was performed via  https://bioinfo.mnhn.fr/abi/public/abgd/  using the Kimura 2-parameter method (Kimura 1980), relative gap width X = 1.5 and intraspecific divergence (P) values ranging from 0.001 to 0.100. For other parameters, the default settings were used.

For species split into more than one BIN, we arranged combined datasets with COI sequences and the nuclear markers EF1α and GADPH. Phylogenetic analysis was made with these concatenated sequences via the Maximum Likelihood method (Felsenstein 1981) and Kimura 2-parameter model (Kimura 1980), using MEGA X version 10.1 (Kumar et al. 2018). Statistical support is given by the bootstrap test with 1000 replicates (Felsenstein 1985).

Specimen details such as collection sites, DNA-Barcode, GADPH and EF1α sequences were uploaded to the BOLD system and are publicly available in the dataset: NZPYR New Zealand Pyraloidea (also see: Suppl. material 1).

## Results

### Genetic distances based on COI barcode sequence using workbench supplied by BOLD

We recovered DNA-barcodes > 500 bp for 440 specimens, with the oldest specimen being from 1993. The number of barcode sequences varies from 1 to 64 sequences per species. BOLD analyses revealed 82 Barcode Index Numbers (BINs) representing 73 morphologically-identified species. These represent 29% of New Zealand Pyraloidea, based on Nuss et. al (2020). For 63 species (86.3%), there was a perfect match between BIN and morphological species identification.

Thirty-four of these BINs already existed on BOLD, with sequences supplied by other BOLD users. We enlarged these BINs with 315 sequences. For six of these BINs, we additionally supplied the species names as they were only identified as Scopariinae. Furthermore, we established 48 new BINs with a total of 125 sequences.

The analysed specimens showed a mean interspecific genetic distance of 12.54% (pairwise analysis, K2P model, n = 61.096 comparisons, SE < 0.01). The mutual comparison of genera showed a mean congenetic distance of 7.99% (pairwise analysis, K2P model, n = 25.274 comparisons, SE < 0.01).

Intraspecific variation showed a mean distance of 0.47%, minimum distance of 0% and a maximum of 6.6% (pairwise analysis, K2P model, comparisons of barcodes with > 500 bp, SE 0.01). The mean distance to the nearest-neighbour (NN) averaged 5.99% with a minimum of 0% and a maximum of 11.04% (pairwise analysis, K2P model, comparisons of barcodes with > 500 bp, SE 0.03) (Tables 1, 2).

Regarding the two most species-rich subfamilies, the specimens of Scopariinae show a mean distance to the nearest-neighbour of 5.4% (pairwise distance, Kimura 2 Parameter, sequences > 500 bp, SE 0.04) with a maximum of 9.0% between *Eudonia trivirgata* and *Antiscopa elaphra* and a minimum of 2.7% between *Eudonia axena* and *Eudonia submarginalis*. With a mean distance of 5.6% in Crambinae (pairwise distance, Kimura 2 Parameter, sequences > 500 bp, SE 0.1), there is a maximum of 11.7% between *Gadira acerella* and *Orocrambus cyclopicus* and a minimum of 0.0% between *Glaucocharis epiphaea* and *Glaucocharis harmonica*.

### Deep intraspecific distances, multiple BIN assignments, BIN and Barcode sharing

There are two BIN assignments which contain two different species each: *G. epiphaea* with *G. harmonica* and *G. helioctypa* with *G. lepidella*. One of these pairs, *G. epiphaea* and *G. harmonica*, even share an identical barcode sequence.

Most of the morphologically-identified species show an intraspecific variation of less than 2%, but seven species (9.6%) show deep variations of up to 6.6%. Six species (8.2%) are spread over more than one BIN. *Orocrambus apicellus*, *Scoparia ustimacula* and *Gadira acerella* appeared each with 2 BINs and *Orocrambus ordishi* and *Orocrambus vulgaris* each with 3 BINs. *Orocrambus ramosellus* appeared in 5 BINs.

The specimens of *Orocrambus vitellus* show a maximum intraspecific distance of 3.76%, but are found in only one BIN. On the contrary, *Gadira acerella* shows a maximum intraspecific distance of 1.96% and is found in two BINs.

Specimens of *Eudonia submarginalis* form five clusters in the barcode Neighbour-Joining analysis (Kimura 2 model, sequences > 500 bp, see Suppl. material 2). Four of these clusters each contain specimens from different sites. One cluster of 20 specimens from Cambrians (Central Otago) is unique as these share an identical barcode sequence and show a distance of 0.81% (pairwise distance, Kimura 2 Parameter, sequences > 500 bp, SE < 0.01) to their nearest group.

The eight specimens of *Orocrambus creneus*, found near Sutton Salt Lake, form a distinct cluster in the barcode Neighbour-Joining analysis (Kimura 2 model, sequences > 500 bp, see Suppl. material 2) compared to one conspecific specimen from Lake Ashburton, which is separated in the barcode Neighbour-Joining analysis with a distance of 1.28% (pairwise distance, Kimura 2 Parameter, sequences > 500 bp, SE 0.01).

**Figure 1.**
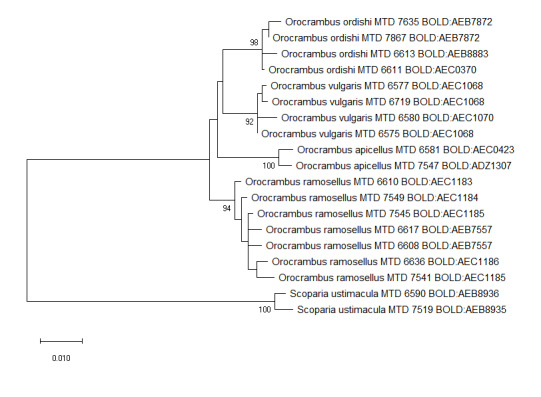
Maximum Likelihood tree using Kimura 2 parameter distance model inferred from EF1α and GADPH sequences (species split into more than one BIN). Bootstrap (1000 replicates) values >= 75% are displayed, branch lengths represent genetic distances between nodes. The scale bar indicates 0.01 K2P distance. The COI BIN number is given for each specimen.

**Figure 2.**
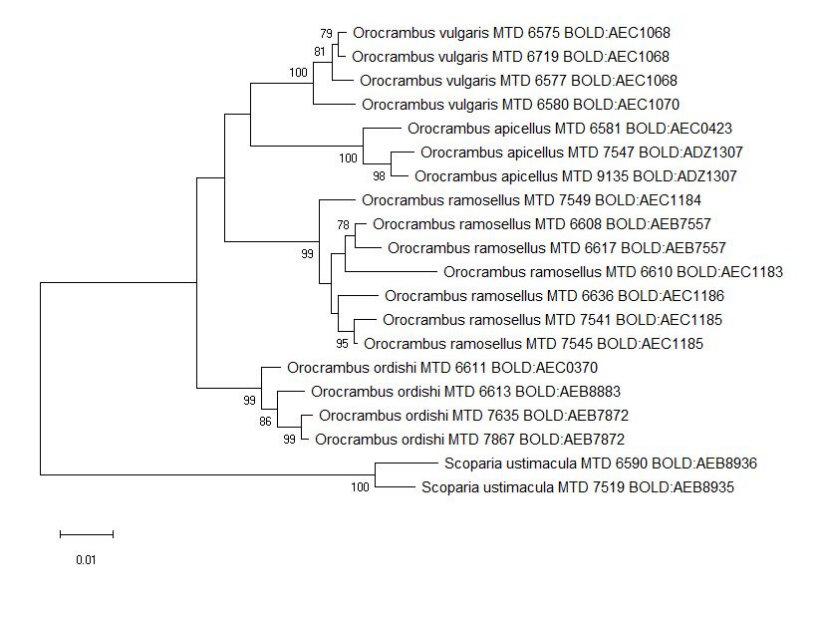
Maximum Likelihood tree using Kimura 2 parameter distance model inferred from COI, EF1α and GADPH sequences (species split into more than one BIN). Bootstrap (1000 replicates) values >= 75% are displayed, branch lengths represent genetic distances between nodes. The scale bar indicates 0.01 K2P distance. The COI BIN number is given for each specimen.

For the species, which appeared in more than one BIN, the concatenated analysis of COI + EF1α + GADPH revealed mean intraspecific distances from 1.12% (*O. ordishi*) to up to 2.0% (*S. ustimacula*) and maximum intraspecific distances from 1.55% (*O. ordishi*) to up to 3.13% (*O. ramosellus*) Table 3Figs 1, 2.

Due to the age of the specimens of *Glaucocharis epiphaea* and *Glaucocharis harmonica* (barcode sharing), as well as of *Gadira acerella*, which is split into two BINs, the amplification and analysis of EF1α and GADPH was not successful.

### ABGD analysis (Automatic barcode gap discovery) in comparison to BIN assignment

**Figure 3.**
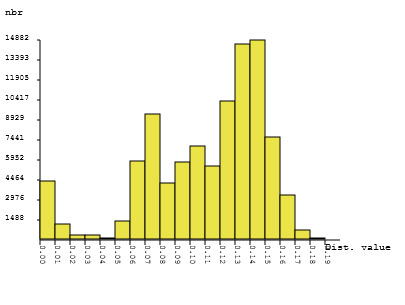
ABGD (Automatic barcode gap discovery) partition analysis of 440 COI sequences of New Zealand Pyraloidea (pairwise distance, Kimura 2 Parameter, sequences > 500 bp, nbr: number of runs) generated via  https://bioinfo.mnhn.fr/abi/public/abgd/  (last access: 10.09.2020)

The automatic barcode gap discovery reveals the presence of a barcode gap at 4% (Fig. 3). For partitioning the dataset, initial partition and recursive partition were used. A total of 440 barcode sequences yielded 88 prospective species following initial partition and 71 prospective species following recursive partition with a 1.3% - 1.7% maximum intraspecific divergence (see Suppl. material 3).

The partition with 88 putative species reveals two OTUs which contain two different species each: *G. epiphaea* with *G. harmonica* and *G. helioctypa* with *G. lepidella*, which is identical to the BIN assignment. Following the partition with 71 putative species, *Eudonia axena*, *Eudonia diphteralis* and *Eudonia submarginalis* together share one OTU Table 4.

## Discussion

From the 250 pyraloid species known from New Zealand (Nuss et al. 2020), 73 morphologically-identified species are included in this study or 29% of the species. Amongst the studied species, there was a perfect match between the BIN assignment and the morphological species identification for 63 species (86.3%). Using the ABGD method, a perfect match between OTU and morphological identification was found for 56 species (76.7%), using initial partition and 62 species (84.9%), using recursive partition. Thus, the level of perfect match depends on the preferred partition.

Considering the accordance between BIN assignment and morphological species identification, former barcode campaigns showed a success rate of about 90% (e.g. Janzen and Hallwachs 2016, Zahiri et al. 2017, Huemer et al. 2020). With 86.3%, our study is close to that value. The success of species identification by barcoding and BIN assignment depends on factors like degrees of relatedness of the tested species and the geographical separation of populations (Elias et al. 2007).

In our survey, there is a collecting bias towards manmade habitats, like pastures and suburban places. Some common species like *Orocrambus flexuosellus* and *Eudonia submarginalis* were found at nearly all study sites. In contrast, uncommon species like *Delogenes limodoxa* and *Glaucocharis elaina* were only found as singletons in one or two protected natural habitats. This imbalance influences the arrangement of OTUs and BINs, so that several BINs are represented by only one specimen.

Barcode sharing has been found for many lepidopteran taxa in previous studies (e.g. Hausmann et al. 2013, Pentinsaari et al. 2014, Bassi and Huemer 2020) and so also in our study with two BINs containing two different species each.

In contrast, six species (8.2%) were split into two to five BINs. For the specimens involved in these BIN splits, the Maximum Likelihood analysis of the concatenated sequences of COI, EF1α and GADPH (Figs 1, 2) provides strongly-supported nodes for those clusters corresponding to our morpho-species identified by characters of wing pattern and genitalia. The branch length seen in the Maximum Likelihood tree is dominated by the COI sequence. Though a strong split into numerous BINs is also found in other lepidopterans, for example, 18 BINs for specimens of the North American erebid *Virbia ferruginosa* (Zahiri et al. 2017) and 22 BINs for specimens of the European gelechiid *Megacraspedus dolosellus* (Huemer et al. 2020), we urge caution as analyses of morphometric and life history data may come to different conclusions.

Several studies suppose a *Wolbachia* infection as a trigger for BIN splitting (e.g. Hausmann et al. 2013, Zahiri et al. 2017). *Wolbachia* infections in New Zealand Pyraloidea are recorded for *Orocrambus enchephorus*, *Eudonia chlamydota*, *Eudonia dinodes*, *Eudonia rakaiensis*, *Eudonia submarginalis*, *Scoparia chalicodes*, *Scoparia rotuella* and *Mnesictena flavidalis* (Woeger et al. 2020). In contrast, no *Wolbachia* infection was recorded for those species with higher sequence variation, leading to multiple BINs per species, i.e. *Orocrambus vulgaris*, *Orocrambus ramosellus*, *Scoparia ustimacula*, *Orocrambus apicellus*, *Orocrambus ordishi* and *Gadira acerella* (Woeger et al. 2020).

The ABGD method results in two different partitions with 88 and 71 putative species, respectively. Depending on the considered partition, the number of OTU sharing and split species is different. Thus, ABGD delivers diverse outcomes and it remains to the user to select and interpret one or more results. Similar to the results obtained with the BIN assignments, we do not see any morphological delimitation supporting different species in these cases of split OTUs.

Several studies have compared results from BIN assignment and ABGD (e.g. Kekkonen and Hebert 2014, Huang et al. 2020). The BIN assignment generates only one result. This might be an advantage as there is no need to make a choice between different ABGD partitions. However, ABGD, as well as BIN assignment, provide several conflicting results, which require further investigation. In most cases, these conflicting results refer to species which are represented by only one specimen (Kekkonen and Hebert 2014, Huang et al. 2020). ABGD is prone to failure when analysing only one or two specimens per species (Puillandre et al. 2011). Likewise, BIN assignments are not stable. With an increase in the number of records, gradual differences of barcode sequences may dissipate and BINs might be lumped together or split (Ratnasingham and Hebert 2013). Nevertheless, barcode-based grouping of specimens can be viewed as the first step within the taxonomic process (Kekkonen and Hebert 2014).

Seventy-one percent of the New Zealand pyraloid species were not available for study due to a limited collecting effort and a bias towards man-made habitats. Further additions to the DNA barcode library will require research on the species that are largely or exclusively restricted to natural habitats and having a restricted area of distribution like *O. sophistis* and *Gadira leucophthalma* (Hoare et al. 2015). Some species are even in urgent need of conservation action, for example, *Gadira petraula, Kupea electilis, O. fugitivellus, O. sophronellus* and *O. ’MacKenzie*’ (Hoare et al. 2015). We support the call by Brian Patrick and the late John S. Dugdale (Patrick and Dugdale 2000) for surveying natural shrub and grassland, coastal areas and lowland forest areas, which hold the most ʽat riskʼ species. Having reference barcodes for declining and endangered species would permit their easy recognition during regular monitoring despite their small body size and rare occurrence in nature, by-passing their time-consuming morphological identification and limiting error rates in identification.

## Supplementary Material

513A5EF8-A6B5-50FC-98C5-B9321DB20B39 10.3897/BDJ.8.e58841.suppl1 Supplementary material 1 Species list. Data type table Brief description Sample IDs, species names, collection sites, BOLD accession numbers File: oo_461141.xlsx https://binary.pensoft.net/file/461141 R. Wöger

0FE3DE52-A5F1-52A4-A57C-32A9487C1714 10.3897/BDJ.8.e58841.suppl2 Supplementary material 2 Neighbour-joining tree Data type Neighbour joining tree Brief description Kimura 2 model, sequences > 500 bp, with species names, collecting ID, subfamily, collecting localities, specimen ID in BOLD database, subfamilies are coloured File: oo_454405.pdf https://binary.pensoft.net/file/454405 R. Wöger

77DEC73B-BA5D-59C9-B5CF-3B24D72B6AB0 10.3897/BDJ.8.e58841.suppl3 Supplementary material 3 Automatic partition results of 440 aligned barcode sequences Data type graph Brief description pairwise distance, Kimura 2 Parameter, sequences > 500 bp; generated via: https://bioinfo.mnhn.fr/abi/public/abgd/ File: oo_454408.png https://binary.pensoft.net/file/454408 R. Wöger

## Figures and Tables

**Table 1.** Species with a COI pairwise distance < 4 % (Kimura 2 Parameter, sequences > 500 bp) to the nearest-neighbor, N = number of examined specimens.

**Species (N)**	**Nearest-neighbour species (N)**	**COI pairwise distance** [%]
*Glaucocharis epiphaea* (1)	*Glaucocharis harmonica* (1)	0.0
*Glaucocharis helioctypa* (1)	*Glaucocharis lepidella* (5)	0.67
*Eudonia axena* (1)	*Eudonia submarginalis* (64)	2.66
*Eudonia diphteralis* (3)	*Eudonia submarginalis* (64)	2.76
*Glaucocharis chrysochyta* (2)	*Glaucocharis selenaea* (2)	3.61
all other species		> 4

**Table 2.** Species with a maximum intraspecific distance > 2.5 % (pairwise distance, Kimura 2 Parameter, sequences > 500 bp), N = number of tested specimens.

**Species (N)**	**mean intraspecific distance** [%]	**max intraspecific distance** [%]
*Orocrambus vulgaris* (16)	2.01	6.6
*Orocrambus ramosellus* (22)	1.44	5.54
*Scoparia ustimacula* (2)	5.52	5.52
*Orocrambus apicellus* (3)	3.16	4.29
*Orocrambus vitellus* (58)	0.73	3.76
*Orocrambus ordishi* (4)	2.19	3.03
*Eudonia submarginalis* (64)	0.86	2.95
all other species		< 2.5

**Table 3.** Mean and maximum intraspecific distances (species split into more than one BIN) analysed with EF1α and GADPH and concatenated sequences (pairwise distance, Kimura 2 Parameter, sequences > 500 bp), N = number of specimens. The particular number of BINs is from COI analysis.

**Species**	**N**	**EF1α**		**N**	**GADPH**		**N**	**concatenated (COI + EF1 α + GADPH)**	
		**mean intrasp. dist.** [%]	**max intrasp. dist.** [%]		**mean intrasp. dist.** [%]	**max intrasp. dist.** [%]		**mean intrasp. dist.** [%]	**max intrasp. dist.** [%]
*O. apicellus* (2 BINs)	2	1.13	1.13	3	0.11	0.17	3	1.44	1.75
*O. ordishi* (3 BINs)	2	0.81	0.81	4	0.25	0.46	4	1.12	1.55
*O. ramosellus* (5 BINs)	5	0.79	1.53	6	0.39	1.07	6	1.72	3.13
*O. vulgaris* (3 BINs)	3	0.62	0.81	3	0.39	0.62	4	1.25	1.82
*S. ustimacula* (2 BINs)	2	0.54	0.54	2	0.93	0.93	2	2.00	2.00

**Table 4.** Species split into more than one OTU/BIN (pairwise distance, Kimura 2 Parameter, sequences > 500 bp). BIN assignment in comparison to the number of putative species following ABGD.

Species	Number of BINs (BOLD)	Putative species (ABGD) partition with 88 OTUs	Putative species (ABGD) partition with 71 OTUs
*O. apicellus*	2	2	2
*O. ordishi*	3	3	1
*O. ramosellus*	5	5	2
*O. vulgaris*	3	3	2
*S. ustimacula*	2	2	2
*G. acerella*	2	2	1
*E. leptalea*	1	2	1
*E. submarginalis*	1	2	1
*O. vitellus*	1	4	1
*P. farinaria*	1	2	1

## Hosting institution

Senckenberg Museum of Zoology, Dresden, Germany
