# Supplementary material for: DNA barcodes for Aotearoa New Zealand Pyraloidea (Lepidoptera)
Source: Biodivers Data J. 2020 Nov 27;8:e58841. doi: 10.3897/BDJ.8.e58841 (PMC7718215; doi:10.3897/BDJ.8.e58841)
Supplement: Supplementary material 2 — Neighbour-joining tree [file bdj-08-e58841-s002.pdf]

BOLD TaxonID Tree

Title	:	Tree Result - NZPYR
Date	:	17-Sep-2020
Data Type	:	Nucleotide
Distance Model	:	Kimura 2 Parameter
Marker	:	COI-5P
Colourization	:	Taxonomy: Subfamily
Label	:	Sample ID
Label	:	Subfamily
Label	:	Taxon
Label	:	Exact Site
Label	:	Barcode Cluster (BIN)
Sequence Count	:	440
Species count	:	73
Genus count	:	18
Family count	:	2
Unidentified	:	0
BIN Count	:	82

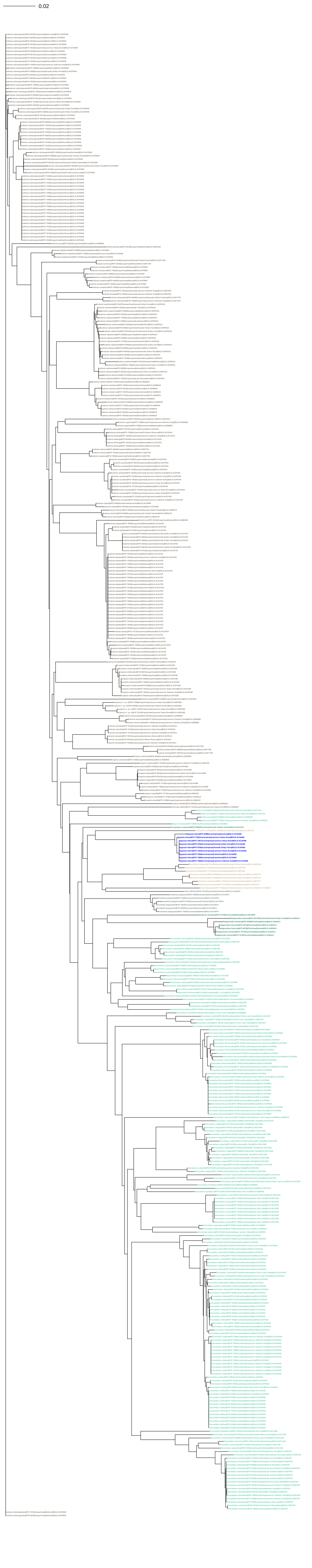
0.02
