# Supplementary figures and images for: DNA barcodes for Aotearoa New Zealand Pyraloidea (Lepidoptera)
Source: Biodivers Data J. 2020 Nov 27;8:e58841. doi: 10.3897/BDJ.8.e58841 (PMC7718215; doi:10.3897/BDJ.8.e58841)

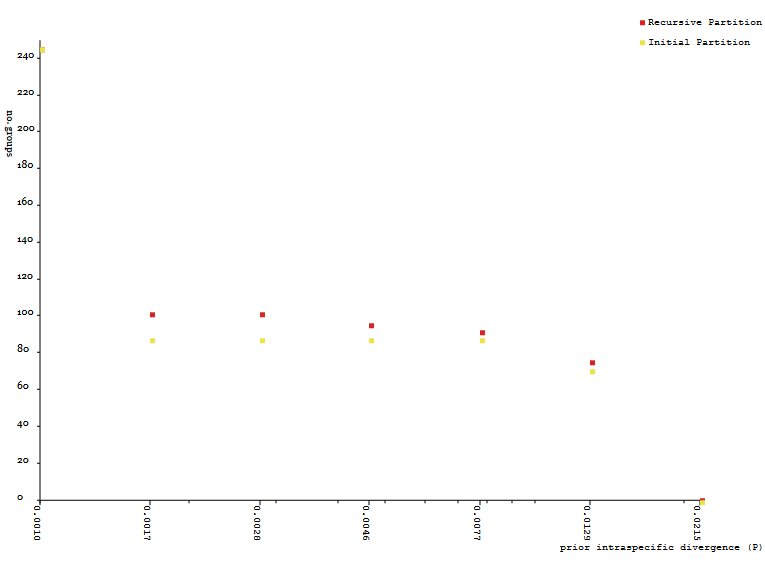

Supplement: Supplementary material 3 — Automatic partition results of 440 aligned barcode sequences [file bdj-08-e58841-s003.png]
